# Real-world evidence of osimertinib in Chinese patients with *EGFR* T790M-positive non-small cell lung cancer: a subgroup analysis from ASTRIS study

**DOI:** 10.1007/s00432-023-04923-8

**Published:** 2023-06-14

**Authors:** Qing Zhou, He-Long Zhang, Li-Yan Jiang, Yuan-Kai Shi, Yuan Chen, Jin-Ming Yu, Cai-Cun Zhou, Yong He, Yan-Ping Hu, Zong-An Liang, Yue-Yin Pan, Wen-Lei Zhuo, Yong Song, Gang Wu, Gong-Yan Chen, You Lu, Cui-Ying Zhang, Yi-Ping Zhang, Ying Cheng, Shun Lu, Chang-Li Wang, Jian-Ying Zhou, Yun-Peng Liu, Jian-Xing He, Jie Wang, Yi-Long Wu

**Affiliations:** 1 Guangdong Lung Cancer Institute, Guangdong Provincial People’s Hospital, Guangdong Academy of Medical Sciences, Guangzhou, China; 2 Department of Oncology, Tangdu Hospital, Fourth Military Medical University, Xi’an, China; 3 Department of Respiratory Medicine, Shanghai Chest Hospital, Shanghai Jiao Tong University, Shanghai, China; 4 Department of Medical Oncology, National Cancer Center/National Clinical Research Center for Cancer/Cancer Hospital, Chinese Academy of Medical Sciences and Peking Union Medical College, Beijing, China; 5 Department of Oncology, Tongji Hospital of Tongji Medical College, Huazhong University of Science and Technology, Wuhan, China; 6 Department of Radiation Oncology, Shandong Cancer Hospital Affiliated to Shandong University, Shandong Academy of Medical Sciences, Jinan, China; 7 Department of Medical Oncology, Shanghai Pulmonary Hospital, Tongji University, Shanghai, China; 8 Department of Respiratory Medicine, Daping Hospital and the Research Institute of Surgery of the Third Military Medical University, Chongqing, China; 9 Department of Oncology, Hubei Cancer Hospital, Wuhan, China; 10 Department of Respiratory and Critical Care Medicine, West China School of Medicine and West China Hospital, Sichuan University, Sichuan, China; 11 Department of Oncology, Anhui Medical University, Hefei, China; 12 Department of Oncology, Xinqiao Hospital, The Third Military Medical University, Chongqing, China; 13 Department of Respiration, General Hospital of Eastern Theater Command of Chinese People’s Liberation Army, Nanjing, China; 14 Cancer Center, Tongji Medical College, Union Hospital, Huazhong University of Science and Technology, Wuhan, China; 15 Department of Respiration, Harbin Medical University Cancer Hospital, Harbin, Heilongjiang, China; 16 Department of Thoracic Oncology, Cancer Center, West China Hospital, Sichuan University, Chengdu, China; 17 Cancer Center, Inner Mongolia Autonomous Region People’s Hospital, Huhhot, China; 18 Department of Thoracic Medical Oncology, Zhejiang Cancer Hospital, Hangzhou, China; 19 Department of Oncology, Jilin Cancer Hospital, Changchun, China; 20 Department of Shanghai Lung Cancer Center, Shanghai Chest Hospital, Shanghai Jiaotong University, Shanghai, China; 21 Department of Lung Cancer, Tianjin Medical University Cancer Institute and Hospital, Tianjin, China; 22 Department of Respiratory Disease, Thoracic Disease Center, First Affiliated Hospital of College of Medicine, Zhejiang University, Hangzhou, China; 23 Department of Medical Oncology, The First Hospital of China Medical University, Shenyang, China; 24 Department of Thoracic Surgery, Guangzhou Institute for Respiratory Health, First Affiliated Hospital of Guangzhou Medical University, Guangzhou, Guangdong China

**Keywords:** Epidermal growth factor receptor, T790M, Non-small cell lung cancer, Osimertinib, Real-world

## Abstract

**Purpose:**

ASTRIS study aimed the largest to evaluate the effectiveness and safety of second- or higher-line osimertinib in patients with advanced/metastatic epidermal growth factor receptor (*EGFR*) T790M mutation-positive non–small cell lung cancer (NSCLC) in the real-world setting. Here we report the results of Chinese patients in ASTRIS study.

**Methods:**

Adults with *EGFR* T790M-positive advanced NSCLC pretreated with EGFR-tyrosine kinase inhibitor (EGFR-TKI), having a WHO performance status score of 0–2 and asymptomatic, stable central nervous system (CNS) metastases were included. All patients received once-daily osimertinib 80 mg orally. The outcomes included investigator-assessed clinical response, progression-free survival (PFS), time-to-treatment discontinuation (TTD), and safety.

**Results:**

A total of 1350 patients were included. Response rate was 55.7% (95% confidence interval [CI] 0.53–0.58). The median PFS and the median TTD were 11.7 months (95% CI 11.1–12.5) and 13.9 months (95% CI 13.1–15.2), respectively. Overall, 389 patients (28.8%) had at least one protocol-specified adverse event (AE); AEs of interstitial lung diseases/pneumonitis-like events and QT prolongation were reported in 3 (0.2%) and 59 (4.4%) patients, respectively.

**Conclusion:**

Osimertinib was effective in Chinese patients with T790M-positive NSCLC who had progressed after first- or second-generation EGFR-TKI in real-word setting and the results were consistent with ASTRIS study overall population and AURA studies. No new safety signals or events were identified.

**Clinical trial number:**

NCT02474355.

## Introduction

Lung cancer is the leading cause of cancer-related deaths worldwide, with a mortality rate of 18% (Sung et al. 2021). In China, lung cancer is the most prevalent cancer, with an estimated 0.82 million new cases in 2020 and 40% of global deaths (Cao et al. 2021). China is expected to witness an increase in magnitude in the lung cancer mortality with 5.07 million deaths in 2040 (Cao et al. 2021). Non-small cell lung cancer (NSCLC) is the most common histologic subtype and accounts for 85% of lung cancer cases in China with one-third of patients having locally or regionally advanced disease at the time of diagnosis (Gan et al. 2021; Govindan et al. 2008). Despite rapid advancements in the diagnosis and treatment of lung cancer, the prognosis for patients with advanced NSCLC remains poor globally, and the 5-year survival rate is less than 10% (Ricciuti et al. 2018).

Epidermal growth factor receptor-tyrosine kinase inhibitors (EGFR-TKIs) are the recommended first-line treatment strategy for patients with advanced NSCLC harboring the *EGFR* sensitizing mutations. However, the majority of the patients develop resistance following treatment with first- or second-generation EGFR-TKIs after a median progression-free survival (PFS) of 9–14 months (Morgillo et al. 2016). The most common mechanism for resistance is the development of “gatekeeper” T790M mutation in exon 20 (converting threonine 790 of the EGFR kinase domain to methionine), which is identified in around 50–70% of resistant cases (Kohsaka et al. 2019; Peng et al. 2021).

Osimertinib is an oral, irreversible EGFR-TKI that selectively inhibits both *EGFR* sensitizing and T790M resistance mutations and has proven to be efficacious in patients with central nervous system (CNS) metastases (Marinis et al. 2019). In a randomized, Phase III AURA3 clinical trial comparing osimertinib with platinum-based doublet chemotherapy in T790M-positive advanced NSCLC patients pre-treated with EGFR-TKI therapy, osimertinib significantly prolonged the median PFS (10.1 vs 4.4 months, 95% confidence interval [CI] 0.23–0.41; P < 0.001) in overall patients and also in patients with CNS metastases (8.5 vs 4.2 months; 95% CI 0.21–0.49) (Mok et al. 2017). The objective response rate (ORR) was also significantly better with osimertinib compared with the chemotherapy (71% vs 31%; P < 0.001). Hence, the AURA3 study established the superiority of osimertinib as the standard of care for patients with confirmed T790M-positive advanced NSCLC after the first-line EGFR-TKI (Mok et al. 2017). Other clinical trials of osimertinib as a second line of treatment for T790M-positive advanced NSCLC, such as AURA2 (Goss et al. 2016), AURA extension (Yang et al. 2017) and a pooled analysis of AURA2/AURA extension trials (Ahn et al. 2019) have also reported a prolonged median PFS of 9.9–12.3 months in overall population. Although the above trials have shown promising efficacy of osimertinib in patients with *EGFR* T790M-positive advanced NSCLC, further evaluation is warranted through a real-world study that will help clinicians gain more effectiveness and safety profile in a large, varied patient population. ASTRIS was aimed at supplementing the existing clinical evidence for the use of osimertinib monotherapy in the *EGFR* T790M-positive advanced NSCLC patients in the real-world setting. Here, we report the results of osimertinib monotherapy in Chinese adults with *EGFR* T790M-positive advanced or metastatic NSCLC from ASTRIS study.

## Materials and methods

### Study design

ASTRIS is an open-label, single-arm, multinational, real-world study. The study was approved by the Institutional Review Board/Ethics Committee and conducted in accordance with the International Conference on Harmonization Good Clinical Practice guidelines, the Declaration of Helsinki, local regulatory requirements, and the policy on bioethics and human biologic samples of AstraZeneca. The study protocol was designed by the sponsor (AstraZeneca, Södertälje, Sweden) in collaboration with investigators and is registered at clinicaltrials.gov (NCT02474355). All patients provided their written informed consent.

### Participants

Chinese adults (≥ 18 years) with locally advanced (stage IIIB) or metastatic (stage IV) NSCLC (based on TNM staging, AJCC 7th edition), WHO performance status (PS) score of 0–2, confirmed T790M mutation and who had received prior EGFR-TKI therapy in any treatment line were included in the study. Patients with asymptomatic or stable CNS metastases were allowed.

Patients with a history of interstitial lung disease (ILD) or symptoms of uncontrolled/severe systemic disease were excluded. The cardiac exclusion criteria were either a mean resting corrected QT interval > 470 ms as per Fredericia formula (Cadogan 2020), factors increasing the risk of arrhythmic events/QTc prolongation, or clinically significant resting electrocardiogram morphological abnormalities.

### Interventions

Patients were given 80 mg osimertinib orally once daily and were continued on treatment if they still receiving clinical benefit, as judged by the investigator.

### Study outcomes and endpoints

The effectiveness and safety data were collected at the treatment visit (every 6 weeks) and termination visit (30 days post last dose). The outcomes included investigator-assessed response rate, PFS, time-to-treatment discontinuation (TTD) and safety profile. This study only collected the incidence of protocol-defined adverse events (AEs), including serious adverse events (SAEs), AEs of special interest (ILD or pneumonitis-like events and QTs prolongation events), and AEs leading to dose modification or discontinuation or death.

### Statistical analysis

All time-to-event outcomes and safety analyses were performed on the full analysis set (FAS) that included all patients receiving at least one dose of osimertinib. The response rate analyses were assessed on the response evaluable set, defined as those patients in the FAS who had at least one documented response assessment by the investigator. Patients who transitioned to a commercial product following national reimbursement were censored at the time of this transition. Time-to-event outcomes (PFS and TTD), including their 95% CIs were estimated using the Kaplan–Meier plot. All AEs and SAEs leading to study drug discontinuation or dose modification were summarized by Medical Dictionary for Regulatory Activities (MedDRA) preferred term (Version 23.1) and Common Terminology Criteria for Adverse Events (CTCAE) class. All statistical analyses were performed using SAS® (SAS Institute, North Carolina) version 9.2 (or later).

## Results

### Patient characteristics

**Fig. 1:**
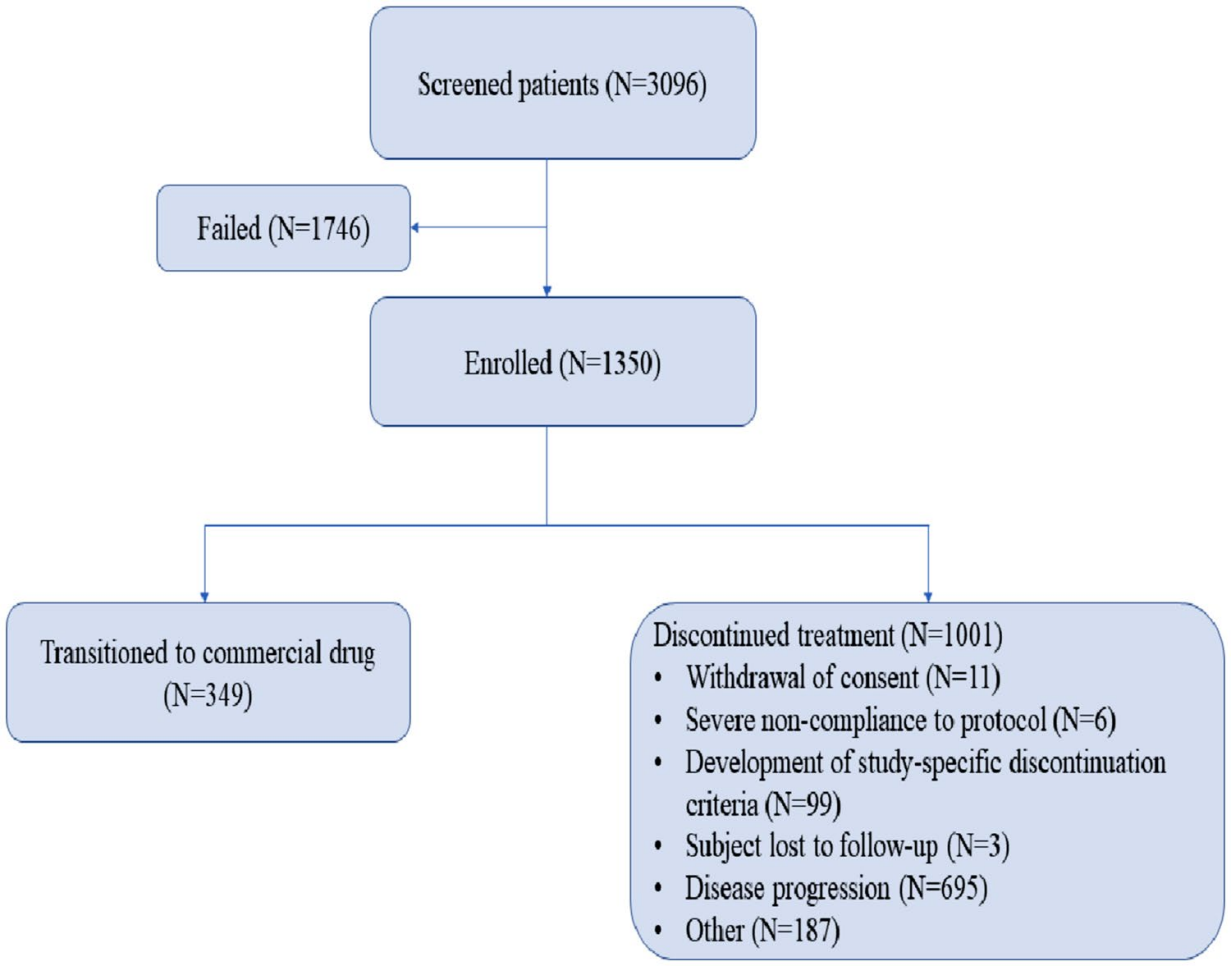
Patient disposition

A total of 1350 Chinese patients were included from September 27, 2016 to September 22, 2017, and received at least one dose of osimertinib. Patient disposition is given in Fig. 1, and the patient demographics and baseline characteristics are summarized in Table 1. Of 1350, 806 (59.7%) patients were female. The median age was 60 years (range 29–87), with 404 (29.9%) patients aged ≥ 65 years. The majority of the patients had stage IV lung cancer (1113; 82.8%) at diagnosis and WHO PS of 1 (973; 72.1%). 774 of 1350 (57.3%) patients had a brain scan available at baseline; 341 of 1350 (25.3%) patients with CNS lesions. All the patients had previously received prior EGFR-TKI treatment; the most commonly used EGFR-TKI was gefitinib in 721 patients (53.4%) followed by icotinib in 371 patients (27.5%) and erlotinib in 286 patients (21.2%). A considerable number of patients had also received prior chemotherapy (651; 48.2%) and radiotherapy (352; 26.1%) (Table 1).

**Table 1:** Baseline demographics and disease characteristics of full analysis set

Characteristics	Osimertinib (N = 1350)
Median age, years (range)	60.0 (29–87)
Age < 65 years, n (%)	946 (70.1)
Age ≥ 65 years, n (%)	404 (29.9)
Gender, n (%)	
Female	806 (59.7)
Male	544 (40.3)
Ethnicity, n (%)	
Asian	1349 (99.9)
Non-Asian	1 (0.1)
WHO performance status, n (%)	
0	268 (19.9)
1	973 (72.1)
2	109 (8.1)
Disease stage at diagnosis, n (%)	1345
Stage 0	3 (0.2%)
Stage IA–IB	61 (4.6%)
Stage IIA–IIB	34 (2.5%)
Stage IIIA	68 (5.1%)
Stage IIIB–IV	1179 (87.7%)
Median duration between diagnosis and enrolment, months, median (range)	23 (1–225)
BM/LM metastases at baseline, n (%)	
Yes	341 (25.3)
BM only	317 (23.5)
LM only	8 (0.6)
Both BM and LM	16 (1.2)
No	433 (32.0)
Not assessed	576 (42.7)
Previous EGFR-TKI^#^, n (%)	
Gefitinib	721 (53.4)
Icotinib	371 (27.5)
Erlotinib	286 (21.2)
Others	35 (2.6)
Prior anticancer therapy^*^, n (%)	
Chemotherapy	651 (48.2)
First line	474 (72.8%)
Second line	266 (40.9%)
≥ Third line	169 (25.9%)
Adjuvant	54 (8.3%)
Neo adjuvant	9 (1.4%)
Palliative	5 (0.8%)
Maintenance	16 (2.5%)
Radiotherapy	352 (26.1)
Other	128 (9.5)
Median duration between last anticancer therapy and enrollment, months, median (range)	0.4 (0–39)

Data are presented as n (%), unless otherwise stated;

BM brain metastases, LM leptomeningeal metastases, EGFR-TKI epidermal growth factor receptor-tyrosine kinase inhibitor, SD standard deviation

^#^Patients may have received more than 1 prior EGFR-TKI

*Patients may have no, one, or more than one previous anti-cancer treatment/radiotherapy/surgery

### *EGFR* T790M testing

*EGFR* T790M molecular testing was performed either on tissue (783; 58%) or blood (567; 42%) samples using different platforms, of which Roche Cobas EGFR assay (Roche Molecular Diagnostics, Inc., CA, USA) was the most commonly used one (1269; 94%). Most of the patients had common mutations (1244, 92.1%), which included T790M either with exon 19 deletion (793, 63.4%) or L858R (451, 36.1%) (Table 2).

**Table 2:** Specimens and platforms used for baseline *EGFR* T790M molecular testing (full analysis set)

Specimen source*/*testing platform	Full analysis set (N = 1350)
Specimen source and testing platform, n (%)	
Tissue	783 (58.0)
Roche Cobas EGFR assay	783 (100.0)
Blood	567 (42.0)
Roche Cobas EGFR assay	486 (85.7)
ddPCR	56 (9.9)
AMOY	16 (2.8)
*EGFR* mutation positive status, n (%)	
T790M	1350 (100)
T790M + Exon 19 deletion	793 (63.4)
T790M + L858R	451 (36.1)
T790M + G719X	6 (0.5)
Mutation combinations, n (%)	
T790M only	89 (6.6)
T790M + common mutations	1244 (92.1)
T790M + uncommon/compound mutations	17 (1.3)
T790M + uncommon mutations	6 (0.4)
T790M + common compound mutations	5 (0.4)
T790M + uncommon compound mutations	6 (0.4)

Common mutations are T790M + Exon 19 Deletion only; T790M + L858R only. Uncommon mutations are T790M + G719X only; T790M + S768I only; T790M + Exon 20 Insertion only. Common compound mutations are T790M + 2 common mutations. Uncommon compound mutations are T790M + 2 or more mutations including at least 1 uncommon mutation

ddPCR droplet digital polymerase chain reaction, EGFR epidermal growth factor receptor

## Effectiveness

### Progression-Free survival

**Fig. 2:**
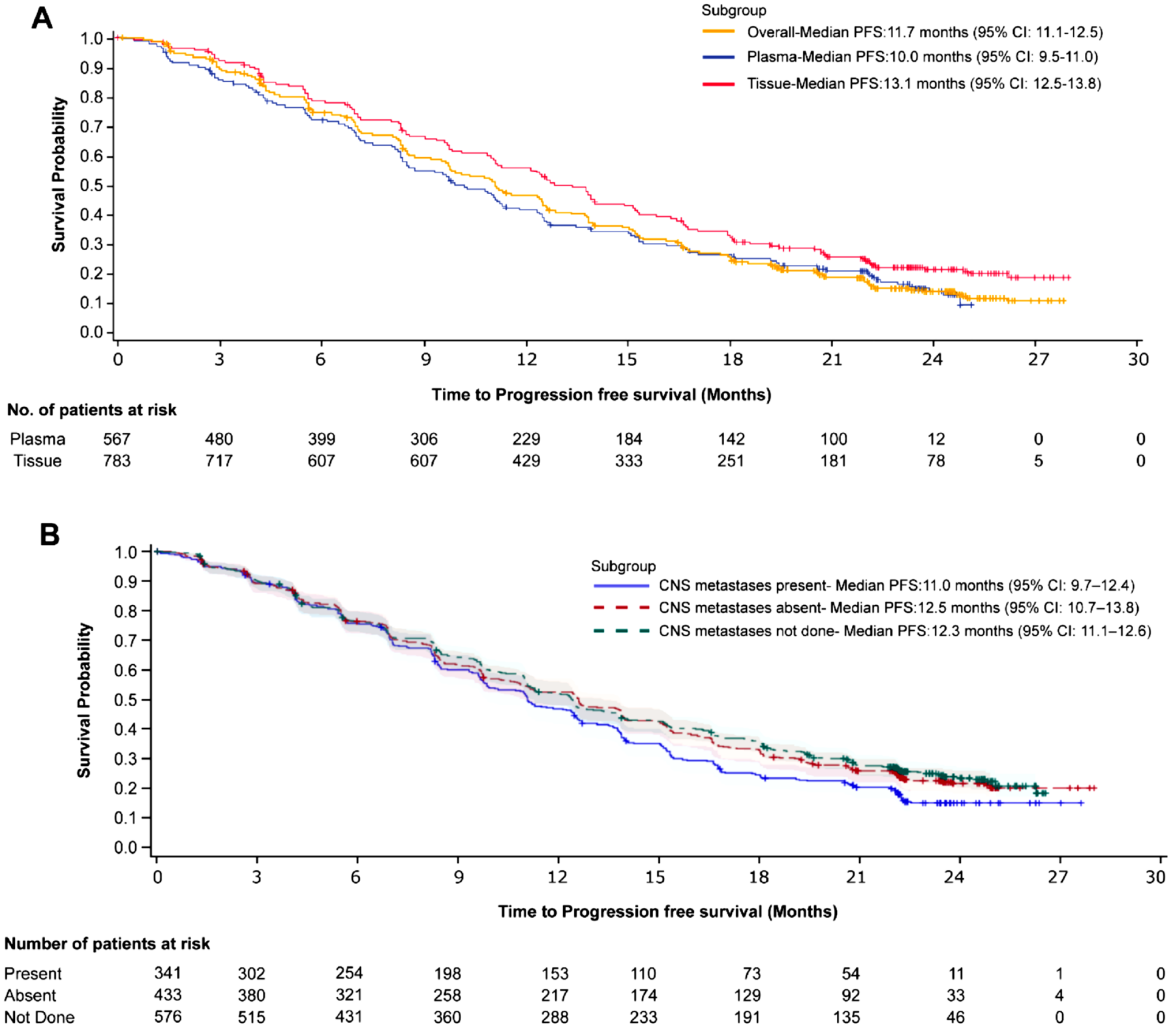
Kaplan-Meir plot for PFS in **a** full analysis set, plasma (Roche Cobas and ddPCR) and tissue (Roche Cobas) and **b** CNS metastases stratified patients. CNS, central nervous system; ddPCR, droplet digital polymerase chain reaction; PFS, progression-free survival

Disease progression or death was reported in 1059 patients (78.4%). The median PFS was 11.7 months (95% CI 11.1–12.5) for the overall population. The median PFS by tissue testing and plasma testing was 13.1 months (95% CI 12.5–13.8) and 10.0 months (95% CI 9.5–11.0), respectively (Fig. 2a). In patients with and without CNS metastases at baseline, the median PFS was 11.0 months (95% CI 9.7–12.4) and 12.5 months (95% CI 10.7–13.8), respectively (Fig. 2b).

### Clinical response rate

Response evaluation was available from 1322 patients; of them, 736 patients (55.7%) had an investigator-assessed best overall response as “responding” (95% CI 52.9–58.4). The response rate in patients with T790M-positive status by tissue testing and plasma testing was 60.4% (95% CI 56.9–63.9) and 48.9% (95% CI 44.6–53.2), respectively. The response rate in patients with and without CNS metastases was 58.2% (95% CI 52.7–63.6) and 57.9% (95% CI 53.0–62.6).

### Time to treatment discontinuation

**Fig. 3:**
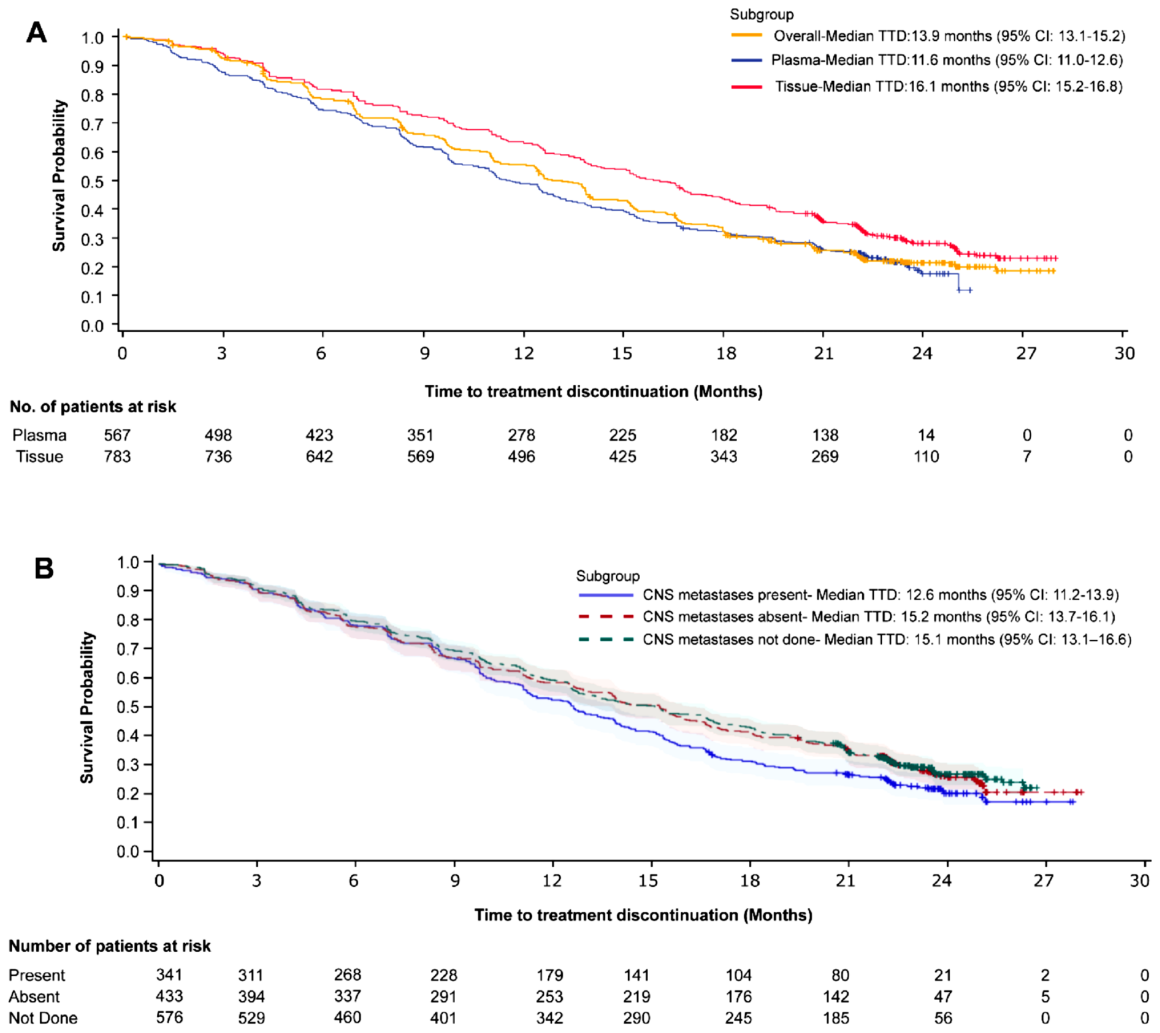
Kaplan-Meir plot for TTD in **a** full analysis set, plasma (Roche Cobas and ddPCR) and tissue (Roche Cobas) and **b** CNS metastases stratified patients. CNS, central nervous system; ddPCR, droplet digital polymerase chain reaction; TTD, time to treatment discontinuation

Of 1350 enrolled patients, 349 patients (25.9%) transitioned to the commercial drug at the time of national reimbursement; the remaining 1001 patients (74.1%) discontinued the treatment, with a median TTD of 13.9 months (95% CI 13.1–15.2). The median TTD in patients with T790M-positive status by tissue testing and plasma testing was 16.1 months (95% CI 15.2–16.8) and 11.6 months (95% CI 11.0–12.6), respectively (Fig. 3a). The prime reason for discontinuation were disease progression (695/1001; 69.4%). The median TTD in patients stratified by the presence or absence of CNS metastases was 12.6 months (95% CI 11.2–13.9) and 15.2 months (95% CI 13.7–16.1), respectively (Fig. 3b).

### Safety

A total of 389 of 1350 (28.8%) patients had at least one protocol-defined AE during the study (Table 3), and 308 of 1350 (22.8%) patients reported an AE with CTCAE grade ≥ 3. Dose modification and discontinuation due to AEs were observed in 124 (9.2%) and 95 (7.0%) patients, respectively. A total of 297 (22.0%) patients had SAEs and were possibly related to osimertinib in 52 (3.9%) patients. Deaths were reported in 92 (6.8%) patients and were possibly related to osimertinib in 14 (1.0%) patients. AEs of special interest were reported in a total of 62 of 1350 (4.6%) patients. Three (0.2%) patients had lung disease/pneumonitis-like events and 59 (4.4%) patients had QTc prolongation events.

**Table 3:** Overall summary of AEs

Adverse events, n (%)	Osimertinib (N = 1350)
Any protocol-specified AE^#^	389 (28.8)
AE leading to dose modification	124 (9.2)
AE leading to discontinuation	95 (7.0)
AE of special interest^*^	62 (4.6)
Lung disease/pneumonitis like event	3 (0.2)
QTc prolongation event	59 (4.4)
Serious AE	297 (22.0)
Treatment related serious AE	52 (3.9)
AE leading to death	92 (6.8)
Treatment related AE leading to death	14 (1.0)
Protocol-specified AEs by preferred term, reported in ≥ 1% of patients	
Electrocardiogram QT prolonged	65 (4.8)
Pneumonia	39 (2.9)
Death	27 (2.0)
Cerebral infarction	18 (1.3)
Pulmonary embolism	16 (1.2)

Data are presented as n (%)

AE adverse event

^#^Safety assessment included serious AEs, AEs leading to dose modification, AEs leading to treatment discontinuation, and AEs of special interest (interstitial lung disease/pneumonitis-like events and QTc prolongation events)

*Interstitial lung disease/pneumonitis-like events and QTc prolongation events

## Discussion

This study aimed to assess the effectiveness and safety of second-line or higher osimertinib in a real-world setting in patients with *EGFR* T790M-positive advanced NSCLC who had received prior EGFR-TKI therapy. Our analysis of Chinese patients is consistent with the ASTRIS global population. OS data in Chinese patients were of low maturity and not reported here. In China, until January 5, 2022, 80/1350 (6%) patients still benefit from the commercial drug and have a duration of osimertinib treatment of more than 4 years from the last patient enrolled.

The median PFS observed in our study was 11.7 months (95% CI 11.1–12.5), which was comparable with that reported in ASTRIS global population (Marinis et al. 2019) (11.1 months; 95% CI 11.0–12.0) and in the previous osimertinib studies, notably AURA3 (Mok et al. 2017) (10.1 months; 95% CI 8.3–12.3), AURA extension (Yang et al. 2017) (12.3 months; 95% CI 9.5–13.8), AURA2(Goss et al. 2016) (9.9 months; 95% CI 8.5–12.3), retrospective study by Peng et al. (Peng et al. 2021) (12.0 months; 95% CI 10.5–13.5), ASTRIS Korean subgroup (Cho et al. 2020) (12.4 months; 95% CI 11.1–13.6), AURA3 Japanese subgroup (Akamatsu et al. 2018) (12.5 months; 95% CI 6.9 to not calculated), and in a Phase III open-label study (Nie et al. 2018) (10.2 months; 95% CI 8.27–9.75) but a recently published Korean real-world study reported a longer PFS (Lee et al. 2023) (14.2 months; 95% CI 13.0–16.4) Several methods are available to detect the presence of *EGFR* T790M from tumor tissue or circulating tumor DNA from plasma (Spence et al. 2021). Tumor tissue is generally preferred and is the current gold standard for detecting resistance mutations (John et al. 2017). For initial *EGFR* T790M testing, however, liquid biopsy is recommended by the National Comprehensive Cancer Network guidelines as an alternative to tissue biopsy (John et al. 2017). The median PFS observed in patients with T790M-positive status by plasma testing in the ASTRIS global study (9.7 months; 95% CI 8.6–10.3), AURA3 study (8.2 months; 95% CI: 6.8–9.7), Korean real-world study (11.0 months; 95% CI 9.0–12.6) was also comparable to our study (10.0 months; 95% CI 9.5–11.0) (Marinis et al. 2019; Mok et al. 2017; Lee et al. 2023). A shorter PFS was observed in plasma *EGFR* T790M-positive patients in the ASTRIS Korean subgroup study (Cho et al. 2020) (6.9 months; 95% CI 2.5–10.9). However, in terms of testing of T790M on tissue, the study reported a median PFS of 13.1 months (95% CI 12.5–13.8) which was in good agreement with the ASTRIS global study (median PFS: 12.7 months; 95% CI 12.4–13.8) (Marinis et al. 2019) and ASTRIS Korean subgroup study (median PFS: 13.6 months; 95% CI 12.0–14.5) (Cho et al. 2020).

The effectiveness was also analyzed by the presence of CNS metastases. The benefit of osimertinib treatment was also found to be extended to the subgroup of Chinese *EGFR* T790M mutation positive NSCLC patients with brain/leptomeningeal metastases reporting a median PFS of 11.0 months (95% CI 9.7–12.4). The findings were consistent with previous reports – AURA extension (Yang et al. 2017) (7.1 months, 95% CI 4.2–12.3), pooled analysis of AURA2/AURA extension (Ahn et al. 2019) (8.2 months, 95% CI 6.9–9.7 months) and AURA3 study (Mok et al. 2017) (8.5 months, 95% CI 6.8–12.3), ASTRIS Korean subgroup study (Cho et al. 2020) (median PFS: 10.8 months; 95% CI 9.5–11.5) and Korean real-world study (Lee et al. 2023) (12.1 months, 95% CI 10.3–14.7). On similar lines, a good response rate of 58.2% (95% CI 52.7–63.6) was achieved in the CNS metastases subgroup in our study which was in line with the AURA extension (Yang et al. 2017) (64%, 95% CI 43–82), pooled analysis of AURA2/AURA extension (Ahn et al. 2019) (59%, 95% CI 51–67) and ASTRIS Korean subgroup study (Cho et al. 2020) (68%, 95% CI 61–74.5). The median TTD in patients with and without CNS metastases was found to be 12.6 months (95% CI: 11.2–13.9) and 15.2 months (95% CI 13.7–16.1), respectively in the present study which was consistent with ASTRIS Korean subgroup study (Cho et al. 2020) (with CNS metastases: 11.2 months, 95% CI 9.4–14.8; without CNS metastases: 14.7 months, 95% CI 12.2–not reached) and Korean real-world study (Lee et al. 2023) (with CNS metastases: 12.5 months, 95% CI 11.0–14.0; without CNS metastases: 15.9 months, 95% CI 14.8–17.0).

Osimertinib was well-tolerated and no new safety signals were observed in our study. The safety profile was similar to that observed in the ASTIRS global population (Marinis et al. 2019), which is an interim analysis of the global study, and AURA clinical studies. The overall incidence rate of adverse events observed in Chinese patients was 28.8% which was comparable to ASTRIS global (30%), and ASTRIS Korean subgroup study (Cho et al. 2020) (31.1%). In these protocol-defined AEs, the most frequent events (> 1% patients) reported in the Chinese population were prolonged electrocardiogram QT (4.8%), pneumonia (2.9%) and death (2.0%). Besides, the incidence of SAEs was also comparable between our study (22%) and ASTRIS global (21%) (Marinis et al. 2019), AURA2 (25%) (Goss et al. 2016) and AURA extension (27%) (Yang et al. 2017) and ASTRIS Korean subgroup study (24.9%) (Cho et al. 2020). Lung disease/pneumonitis-like event and QTc prolongation were the AEs of special interest observed at a rate of 0.2% and 4.4%, respectively in our study compared to 1% and 3%, respectively seen in ASTIRS global (Marinis et al. 2019), 2% and 6% respectively in AURA2 (Goss et al. 2016), 4% each in AURA3 (Mok et al. 2017) study and 1.7% and 1.5%, respectively in ASTRIS Korean subgroup study (Cho et al. 2020).

Although our study gives a better understanding of the effectiveness and safety aspects of osimertinib in a real-world scenario in a Chinese setting complementing the results of a global setting, certain limitations must be taken care of before drawing any conclusion. Considering the fact that due to many patients transitioning to commercial supply following national reimbursement as per ASTRIS protocol, the follow-up was incomplete and the exposure may have been underestimated. Also, the response rates were investigator-assessed as per institutional standards. Not all patients were assessed at baseline for the CNS metastases and the types of samples used and the availability of the type of testing methods for T790M at different study sites could also have introduced bias in the study results.

## Conclusion

The real-world effectiveness and safety results in Chinese patients supporting osimertinib as a standard second-line treatment in patients with T790M-positive advanced NSCLC.

## Data Availability

The datasets that were used and analysed during the current study are available from the corresponding authors on reasonable request.
